# Thoracic Kidney: Extremely Rare State of Aberrant Kidney

**DOI:** 10.1155/2015/672628

**Published:** 2015-08-02

**Authors:** Mahdi Khoshchehreh, Omalbanin Paknejad, Mehrdad Bakhshayesh-Karam, Marzieh Pazoki

**Affiliations:** ^1^Department of Preventive Medicine, Keck School of Medicine, University of Southern California, 2001 N. Soto Street, Los Angeles, CA 90033, USA; ^2^Department of Medicine, Division of Cardiology, University of California, Irvine, Irvine, CA, USA; ^3^Pulmonology Department, Shariati Hospital, Tehran, Iran; ^4^National Research Institute of Tuberculosis and Lung Disease, E19575/154 Darabad, Shaheed Bahonar Avenue, Tehran 19556, Iran; ^5^Pulmonology Department, Sina Hospital, Tehran, Iran

## Abstract

The thorax is the rarest place among all forms of renal ectopia. We report a rare case of an unacquired thoracic kidney. Only about 200 cases of the thoracic kidney have ever been reported in medical literature worldwide. In this paper we present the rarest form of nontraumatic nonhernia associated, truly ectopic thoracic kidney. The differential diagnosis and management options and classification of this rare form of aberrant kidney are discussed.

## 1. Introduction

Urinary system anomalies affect approximately 10% of population [1]. The thorax is an extremely rare state of aberrant kidney, accounting for less than 5% of renal ectopia. The thoracic kidney is reported more often in males and usually occurs on the left side [1].

In contrast to pelvic kidneys and other renal ectopia cases, the majority of patients with an intrathoracic kidney are asymptomatic and their kidney functions are almost always normal [2]. Therefore, most cases are discovered incidentally as a mass on a chest radiograph. Further studies, like computed tomography (CT) or intravenous urography may be capable of differentiating it from other intrathoracic masses. We present the CT and ultrasonographic findings of an adult male with this rare anomaly.

## 2. Case Report

A 32-year-old man presented with cough and shortness of breath for 4 months, which became noticeable at the start of the winter. He was not on medication, and he had an uneventful life.

**Figure 1:**
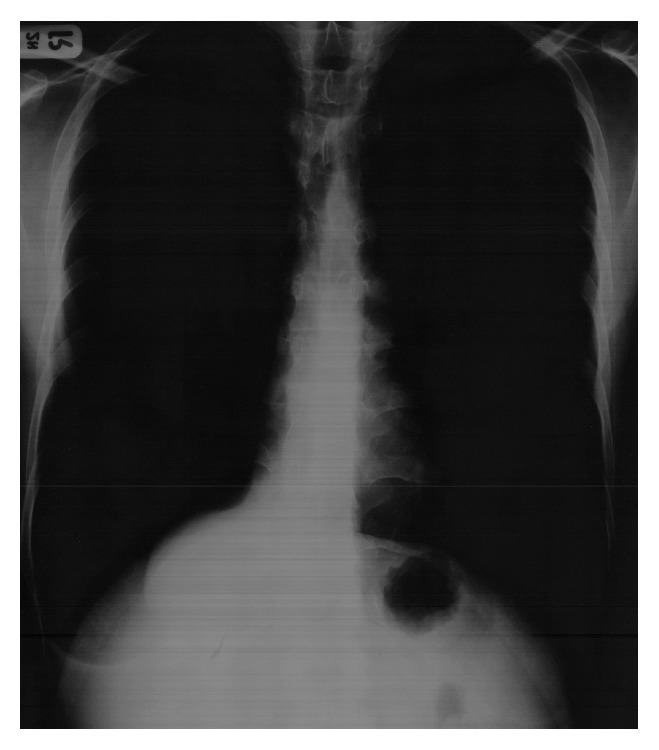
32-year-old man, diagnosed for ectopic kidney; chest X-ray demonstrated hyperinflation of both lungs, with round soft tissue density in the cardiophrenic angle, which does not silhouette the cardiac shadow.

**Figure 2:**
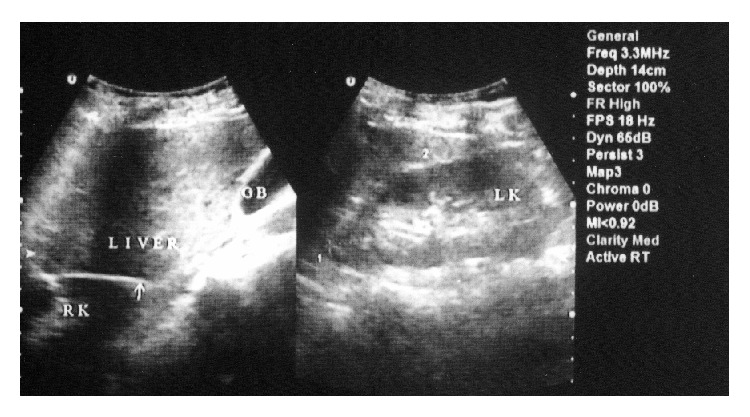
32-year-old man, diagnosed for ectopic kidney; ultrasonographic evaluation revealed an ectopic right kidney above the right hemidiaphragm. Both kidneys have normal size, echo pattern, and cortical thickness.

**Figure 3:**
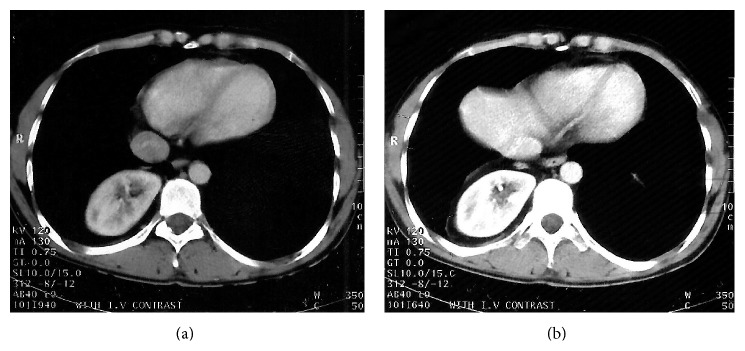
32-year-old man with thoracic ectopic kidney; an axial contrast enhanced CT scan of the revealed right kidney is in the lower portion of the right hemithorax.

Physical examination was normal, including normal breathing sounds all over the lung without any abnormal resonance and symmetrical expansion of the thorax. Laboratory investigations were within normal limits. A chest roentgenogram (Figure 1) showed hyperinflation of both lungs, with round soft tissue density in the cardiophrenic angle, which does not silhouette the cardiac shadow. Upon ultrasonic examination (Figure 2), the right kidney was ectopic and was located above the right hemidiaphragm. Both kidneys had normal size, echo pattern, and cortical thickness. CT scan (Figures 3(a) and 3(b)) suggested that the right kidney was in the lower portion of the right hemithorax. Respiratory function tests revealed an obstructive pattern, which was reversible with bronchodilator therapy, compatible with asthma.

## 3. Discussion

Thoracic kidney is the rarest form of renal ectopia, while only about 200 cases have been reported and published in medical literature [3]. Most cases are discovered as a result of investigation of a mass revealed by chest radiographies. In this condition, other abdominal organs have not advanced into the chest cavity, in contrast to an aberrant kidney that is secondary to a congenital or traumatic diaphragmatic hernia. The aberration is found on the left in 62% of cases and is infrequently bilateral (2%), with large male predominance [1, 2].

Thoracic kidney has been described in four basic categories: (i) true ectopia; (ii) diaphragm eventration; (iii) diaphragmatic hernia; and (iv) traumatic diaphragm injuries [4]. True ectopia, may result from an accelerated ascent of embryonic metanephros or delayed closure of the diaphragm. Although it is extremely rare anomaly, association with thoracic kidney has been reported. The adrenal gland is in the normal position in a large number of patients [2].

Imaging studies, including CT and intravenous urography, are capable of differentiating aberrant kidneys from other intrathoracic masses [5]. MRI-urography has been suggested as the next step of choice when the ultrasound findings are equivocal [5, 6]. During evaluation of a patient with thoracic kidney, searching for possible associated anomalies is mandatory, since some thoracic kidneys have been reported in fetal demise and early neonatal death due to severe multiple congenital anomalies [2, 7].

In our case, since the patient's history did not include a traumatic accident, the radiographic findings were compatible with a diagnosis of ectopic kidney. The bottom line is that although thoracic kidney is rare, it must be considered in patients with an intrathoracic mass. Frequently, it does not affect renal function and therefore it does not require any further intervention.
